# Folic acid protects against arsenic-mediated embryo toxicity by up**-**regulating the expression of Dvr1

**DOI:** 10.1038/srep16093

**Published:** 2015-11-05

**Authors:** Yan Ma, Chen Zhang, Xiao-Bo Gao, Hai-Yan Luo, Yang Chen, Hui-hua Li, Xu Ma, Cai-Ling Lu

**Affiliations:** 1 Graduate School of Peking Union Medical College, Beijing, China.; 2 Department of Genetics, National Research Institute for Family Planning, Beijing, China.; 3 MOE Key Laboratory of Bioinformatics, TNLIST Bioinformatics Division & Center for Synthetic and Systems Biology, Tsinghua University, Beijing, China.; 4 Department of Nutrition and Food Hygiene, School of Public Health, Dalian Medical University, Dalian, China.; 5 Department of Cardiology, Institute of Cardiovascular Disease, First Affiliated Hospital of Dalian Medical University, Dalian, China

## Abstract

As a nutritional factor, folic acid can prevent cardiac and neural defects during embryo development. Our previous study showed that arsenic impairs embryo development by down-regulating Dvr1/GDF1 expression in zebrafish. Here, we investigated whether folic acid could protect against arsenic-mediated embryo toxicity. We found that folic acid supplementation increases hatching and survival rates, decreases malformation rate and ameliorates abnormal cardiac and neural development of zebrafish embryos exposed to arsenite. Both real-time PCR analysis and whole in-mount hybridization showed that folic acid significantly rescued the decrease in Dvr1 expression caused by arsenite. Subsequently, our data demonstrated that arsenite significantly decreased cell viability and GDF1 mRNA and protein levels in HEK293ET cells, while folic acid reversed these effects. Folic acid attenuated the increase in subcellular reactive oxygen species (ROS) levels and oxidative adaptor p66Shc protein expression in parallel with the changes in GDF1 expression and cell viability. P66Shc knockdown significantly inhibited the production of ROS and the down-regulation of GDF1 induced by arsenite. Our data demonstrated that folic acid supplementation protected against arsenic-mediated embryo toxicity by up-regulating the expression of Dvr1/GDF1, and folic acid enhanced the expression of GDF1 by decreasing p66Shc expression and subcellular ROS levels.

The contamination of drinking water by inorganic arsenic is a great threat to public health in many countries. Chronic arsenic exposure increases the risk for and incidence of cancer, cardiovascular disease, developmental and reproductive problems^1^,^2^. It was reported that in utero arsenic exposure can cause congenital heart disease in an offspring whose mother consumed contaminated water during pregnancy^3^. Drinking water with high arsenic levels is a risk factor for neonate deformity, including types of nervous system deformities, limb deformities and congenital heart disease, in Chinese populations^4^. Animal studies showed that arsenic induces preimplantation developmental retardation as well as postnatal growth retardation and malformation^5^,^6^.

Oxidative stress has been identified as an important mechanism of arsenic toxicity^7^. Arsenic-induced ROS-mediated apoptosis has been well documented in many cell lines^8^,^9^. Recent studies reported that arsenite stimulates nicotinamide adenine dinucleotide phosphate (NADPH) oxidase present in the plasma membrane of vascular endothelial cells and vascular smooth muscle cells to increase the generation of reactive oxygen species (ROS), such as superoxides and hydrogen peroxide^10^,^11^. P66Shc participates in the production of mitochondrial ROS by serving as a redox enzyme that oxidizes cytochrome c, thus generating proapoptotic H_2_O_2_ in response to specific stress signals^12^. Our previous study showed that arsenite induces a severe redox imbalance by decreasing glutathione levels and increasing ROS levels through p66Shc, which induces apoptosis by activating the cytochrome c-caspase pathway^13^. Both p66shc knockdown and the antioxidant N-acetylcysteine (NAC) improve the developmental competence of arsenite-exposed embryos *in vitro* by increasing the resistance to oxidative stress^13^,^14^. Trinei *et al.* reported that p66Shc acts as a downstream target of the tumour suppressor p53 and is indispensable for the ability of stress-activated p53 to induce the elevation of intracellular oxidants, cytochrome c release and apoptosis^15^.

Folic acid, a water-soluble vitamin B, cannot be synthesized *in vivo* but is instead absorbed from green leafy vegetables and citrus fruits^16^. It is well known that dietary folic acid supplementation reduces neonatal mortality from neural tube disorders^17^. Several studies have shown that periconceptional multivitamins containing FA may reduce the risk of congenital heart defects^18^,^19^. Dietary supplementation with FA prevented ethanol-induced cardiac birth defects in mice^20^. Our previous study showed that folic acid protected against selenite-induced embryo toxicity, including cardiac and neural defects, in zebrafish^21^. Han *et al.* reported that folic acid involved the canonical Wnt pathway to rescue lithium-induced vertebrate cardiac anomalies^22^. Folic acid has recently attracted much attention as a nutritional factor that influences arsenic-induced toxicity. Xu *et al.* reported that folic acid supplementation attenuates arsenic-induced toxicity and apoptosis in hepatocytes^23^. Folic acid treatment could protect SWV/Fnn mouse fibroblasts from sodium arsenite cytotoxicity^24^. Our previous study showed that embryonic arsenic toxicity during zebrafish development is characterized by pericardium oedema, circulation failure, looping failure and neurodevelopmental confusion^25^. Further analysis indicated that zebrafish Dvr1 (decapentaplegic and Vg-related-1), a mammalian homologue of GDF1 (growth differentiation factor 1) related to the formation of the left–right axis, was involved in arsenic-mediated embryo toxicity^26^. However, whether folic acid can rescue the developmental toxicity of arsenic in zebrafish embryos remains elusive.

In this study, we evaluated the effect of folic acid on arsenic-induced embryonic toxicity in zebrafish and found that folic acid could protect the development of the cardiovascular and nervous systems against arsenic toxicity. Folic acid decreased oxidative stress and enhanced the expression of Dvr1/GDF1 by inhibiting the expression of the oxidative stress adaptor p66Shc. This study suggested that folic acid could be an effective protector against arsenic toxicity by up-regulating Dvr1 activity.

## Results

### Folic acid increases hatching and survival rates and decreases the malformation rate of embryos exposed to arsenite

**Figure 1:**
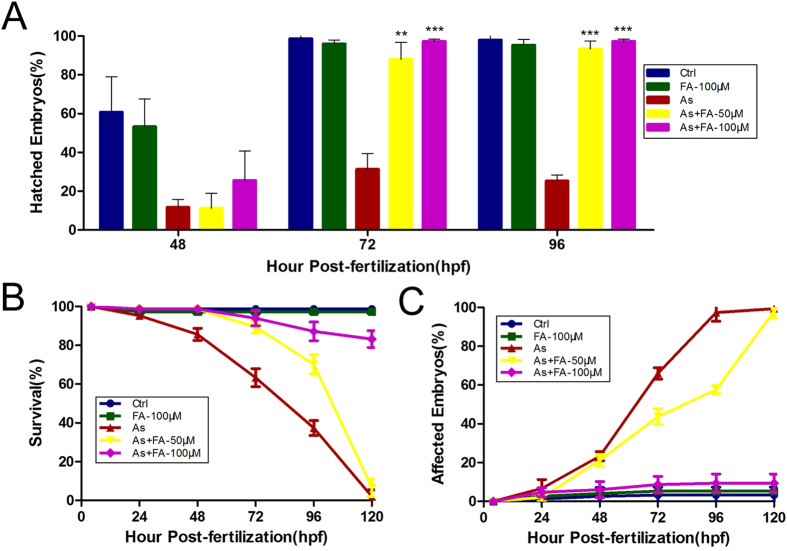
Folic acid increases hatching and survival rates and decreases the malformation rate of embryos exposed to arsenite. (**A**) Histogram indicates the hatchability of embryos rescued by folic acid against hour post-fertilization (hpf). Statistically significant differences from the arsenite-treated group are indicated by asterisks. ^**^p < 0.01, ^***^p < 0.001 vs arsenite-treated group, ANOVA. (**B**) The percentages of survival are plotted against the hour post-fertilization (hpf). (**C**) The percentages of affected embryos are plotted against the hour post-fertilization (hpf). Value are presented as mean ± S.E. Ctrl, control; FA, folic acid; As, arsenite. (Each treatment was replicated three times; n = 50 for each treatment).

Our previous study showed that arsenite delays hatching and induces abnormal morphology and mortality^25^. To assess whether folic acid could rescue arsenic-mediated embryo toxicity, we treated zebrafish embryos with 2 mM arsenite, different doses of folic acid, and their combination at 4 hpf. The hatching, survival and malformation rates were observed at different time points. As illustrated in Fig. 1A, the hatching rates of the control and folic acid-treated (100 μM) group were more than 50% at 48 hpf. Only 10% of the embryos hatched by 48 hpf in the groups treated with arsenite. The hatching rate of the group co-treated with arsenite and 100 μM folic acid increased slightly. At 72 and 96 hpf, the hatching rate was approximately 98% in the control and folic acid-treated groups but was only 30% in the group treated with arsenite. Co-treatment with 50 or 100 μM folic acid significantly ameliorated the decrease in the hatching rate mediated by arsenite. Furthermore, the hatching rate in the group co-treated with arsenite and 100 μM folic acid was approximately the same as that in the control group.

As shown in Fig. 1B, there was no significant difference in the survival of embryos in all the groups before 24 hpf. The survival rate sharply decreased in the arsenite-treated group from 48 to 120 hpf. Co-treatment with 50 or 100 μM folic acid obviously increased the survival rate of the arsenite-treated group from 48 to 96 hpf. Moreover, the survival rate after folic acid supplementation with 100 μM was higher than that with 50 μM folic acid. However, the protective effect of folic acid decreased from 48 to 120 hpf. The survival rate after folic acid supplementation in the 50 μM group decreased to a similar level as that in the arsenite-treated group at 120 hpf. Notably, supplementation with 100 μM folic acid rescued the survival rate of embryos exposed to arsenite quite well at 120 hpf.

In Fig. 1C, arsenite treatment led to an obvious increase in the malformation rate during development from 48 to 120 hpf compared with the control group. Co-treatment of embryos with arsenite and 100 μM folic acid maintained normal embryo development at these time points, which was almost consistent with the control group. Co-treatment with 50 μM folic acid partly rescued the abnormal embryos caused by arsenite treatment from 48 to 96 hpf. These data indicated that folic acid alleviated the embryo toxicity of arsenite in zebrafish to some extent.

### Folic acid protects against arsenite-inducing cardiac defects

**Figure 2:**
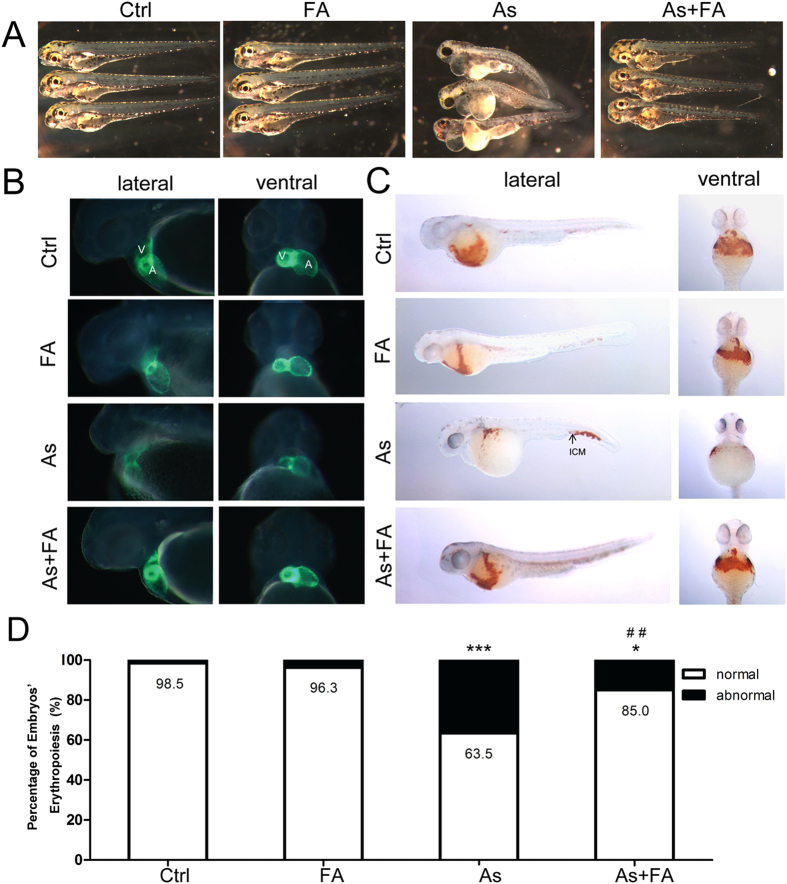
Folic acid protects against arsenite-inducing cardiac defects. (**A**) Embryos exposed to arsenic caused abnormal development, especially pericardial oedema, but rescued by folic acid supplement at 72 hpf. (**B**) The phenotypes of heart looping and chambers were observed using Tg (cmlc2:gfp) transgenic embryos. Representative lateral and ventral view fluorescence micrographs showing ventricle (V) and atrium (A) are showed at 48 hpf. (**C**) and (**D**) O-dianisidine staining was used to detect distribution and number of erythroid cells in the control and treated embryos at 48 hpf. Erythroid cells were found on the yolk sac and intermesiate cell mass (ICM). Arrowhead is positioned to note ICM. Graphs illustrate the percentage of embryos with normal erythropoiesis in control and treated groups embryos at 48 hpf. ^*^p < 0.05, ^***^p < 0.001 vs control group; ^##^p < 0.01 vs arsenite treated group. The data represent the mean ± S.E. Ctrl, control; FA, folic acid; As, arsenite. (Each treatment was replicated three times; n = 50 for each treatment).

The embryos treated with arsenite exhibited a spectrum of developmental abnormalities, including pericardial oedema, looping failure, dorsal curvature and a flat head. The malformation of arsenite-exposed embryos was rescued by folic acid supplementation, with similar results as those in the control and folic acid-treated groups (Fig. 2A). Subsequently, we checked the development of chambers and cardiac looping using Tg (cmlc2:GFP) transgenic zebrafish embryos. Arsenite-treated embryos showed a tubular heart with a smaller ventricle and looping failure between the two chambers. Embryos exposed to arsenite and folic acid exhibited increased ventricle development and cardiac looping compared to those treated with arsenite (Fig. 2B). To further investigate the effect of folic acid on embryo haemoglobin production, we detected erythropoiesis by o-dianisidine staining. Erythroid cells were visible in the control and folic acid-treated groups at 48 hpf but disappeared in the cardiovascular system and singularly aggregated in the ICM (intermediate cell mass) in embryos treated with arsenite. However, co-treatment with arsenic and folic acid restored the reduced erythropoiesis induced by arsenic compared to treatment with arsenic alone (Fig. 2C). In addition, the percentage of embryos with normal erythropoiesis was decreased to 63.5% in the arsenite-treated group. The percentage of embryos with normal erythropoiesis in the arsenic and folic acid co-treatment group was significantly improved to 85% (Fig. 2D).

### Folic acid protects the axon scaffold and CNS against arsenic toxicity

**Figure 3:**
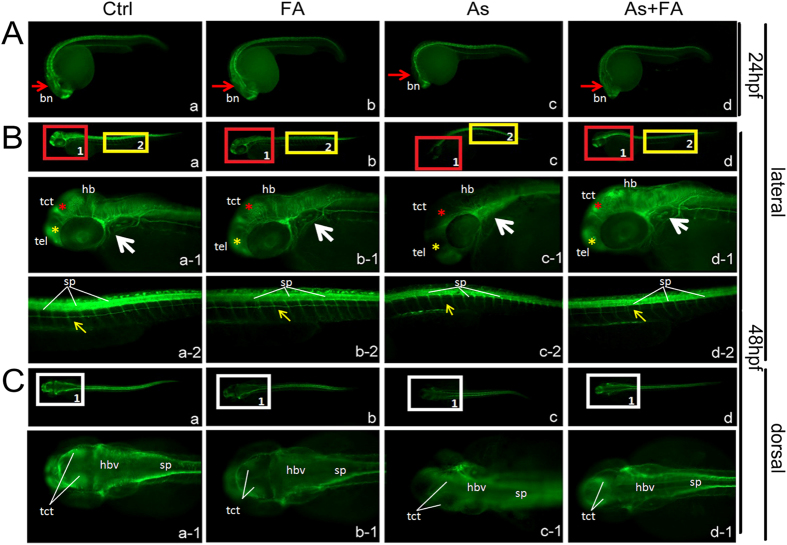
Folic acid protects the axon scaffold and CNS against arsenic toxicity. The development of neural system was indicated by immunostaining of α-AT (green fluorescence). (**A**) Lateral view of 24 hpf in the control group (a) and groups treated with FA (b), As (c), and their combination (d), respectively. (**B**) Lateral view of 48 hpf in the control (a) and groups treated with FA (b), As (c), and their combination (d), respectively. The boxed area in each group were magnified as (a-1), (a-2), (b-1), (b-2), (c-1), (c-2), (d-1), and (d-2), respectively, to provide clearer views of nerve growth in the brain and trunk. Red asterisks indicate the nerve branches in tectum and tectal ventricle; Yellow asterisks indicate the nerve branches in telencephalon; White arrowheads are positioned to note the nerve branches in otic vesicle; Yellow arrowheads indicate the motor neuron development in trunk. (**C**) Dorsal view of 48 hpf in the control (a) and groups treated with FA (b), As (c), and their combination (d), respectively. The boxed area in each group were magnified as (a-1), (b-1), (c-1) and (d-1), respectively, to provide clearer views of nerve growth in the brain. Views in (**B**) are lateral with anterior to the left; views in (**C**) are dorsal with anterior to the left. Each treatment was replicated three times. bn, brain; hb, hindbrain; hbv, hindbrain ventricle; sp, spinal cord; tel, telencephalon; tct, tectum and tectal ventricle. Ctrl, control; FA, folic acid; As, arsenite.

To test the ability of folic acid to rescue the neural development of embryos after exposure to arsenite, we detected acetylated α-tubulin, a pan-axonal marker, by immunostaining at 24 and 48 hpf. At 24 hpf, embryos in the arsenite-treated group exhibited a reduction in these signals in the brain area compared with the control group, the folic acid-treated group and the arsenite and folic acid co-treatment group (Fig. 3A). At 48 hpf, arsenite treatment resulted in a chaotic arrangement of the axon scaffold and the irregular distribution of motor neuron axons between segments (Fig. 3B-c) in contrast to the control group (Fig. 3B-a) and the folic acid-treated group (Fig. 3B-b). Similarly, the neural patterning in the hindbrain area of zebrafish embryos treated with arsenite was vague (Fig. 3C-c) compared with the control group (Fig. 3C-a) and the folic acid-treated group (Fig. 3C-b), which exhibited normal patterning. Supplementation with folic acid rescued the deleterious effect of arsenite on neural development (Fig. 3B-d,C-d). The embryos in the co-treatment group exhibited generally increased axons in all areas of the brain, and the axon tracts in the caudal region did not appear to be affected. These data indicated that folic acid could protect the axon scaffold and CNS against arsenite-mediated embryo toxicity.

### Folic acid protects against arsenic-induced developmental defects by up-regulating the expression of Dvr1/GDF1

**Figure 4:**
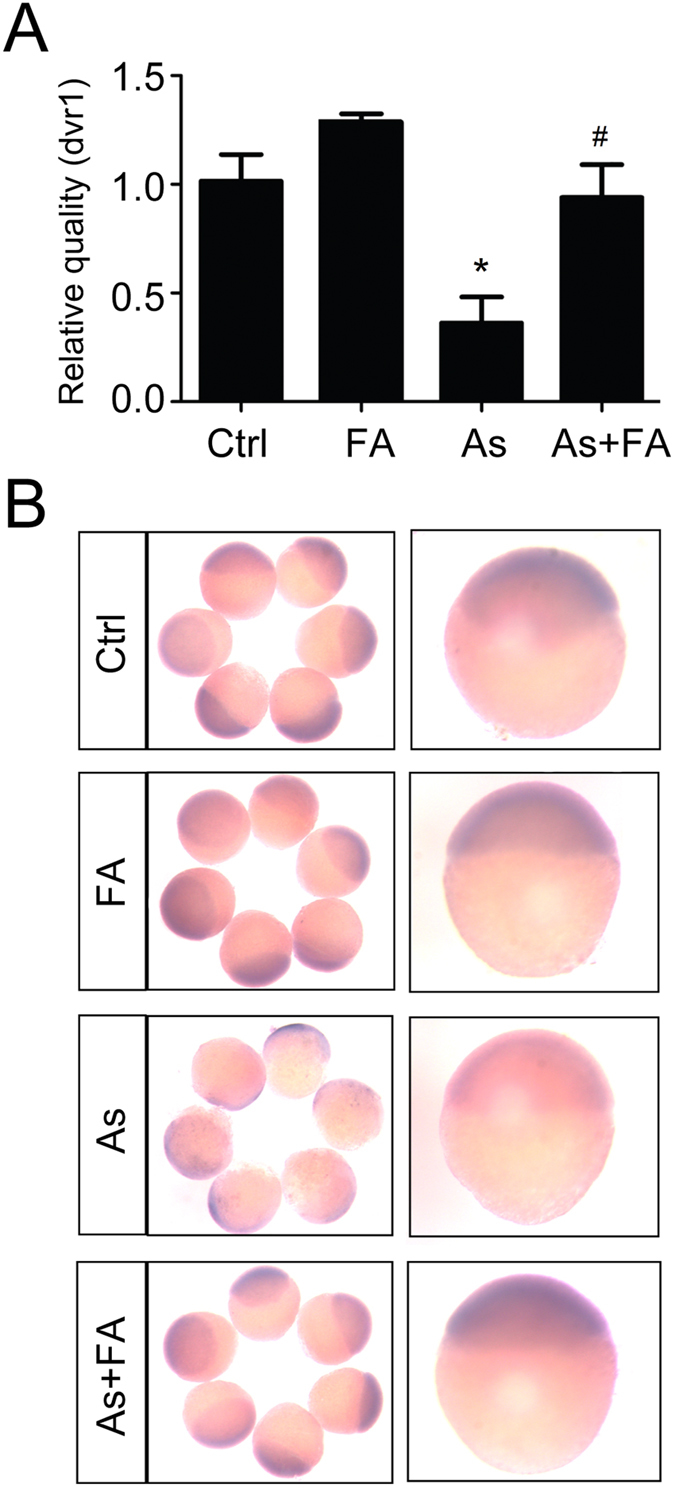
Folic acid protects against arsenic-induced developmental defects by up-regulating the expression of Dvr1/GDF1. (**A**) The mRNA level of Dvr1 in control and treated groups embryos at 6 hpf were measured by quantitative real-time PCR. ^*^p < 0.05 vs control group; ^#^p < 0.05 vs arsenic group. The data represent the mean ± S.E. (**B**) Dvr1 expression pattern in control and treated groups embryos at 6hpf by whole-mount *in situ* hybridization. Ctrl, control; FA, folic acid; As, arsenite.

Our previous research showed that arsenic impairs embryonic development by down-regulating Dvr1 (a homologue of mammalian GDF1) expression in zebrafish^26^. To investigate the possible mechanism of folic acid protection against arsenite-induced toxicity, Dvr1 was examined in zebrafish embryos treated with arsenite, folic acid and their combination at 4 hpf (sphere stage). Real-time PCR analysis showed that the level of Dvr1 in arsenic-treated embryos was markedly lower compared to that in the control groups at 6 hpf (shield stage), which is consistent with our previous results. However, co-treatment with folic acid and arsenite led to a significant increase in Dvr1 mRNA levels compared with arsenite-only treatment; the levels were approximately the same level as those in the control group (Fig. 4A). This result was further supported by the results of whole-mount *in situ* hybridization (Fig. 4B). These data suggested that folic acid protects against arsenic-induced developmental defects by up-regulating the expression of Dvr1.

### Folic acid up-regulates cell viability and the expression of GDF1 in arsenite-exposed HEK293ET cells

**Figure 5:**
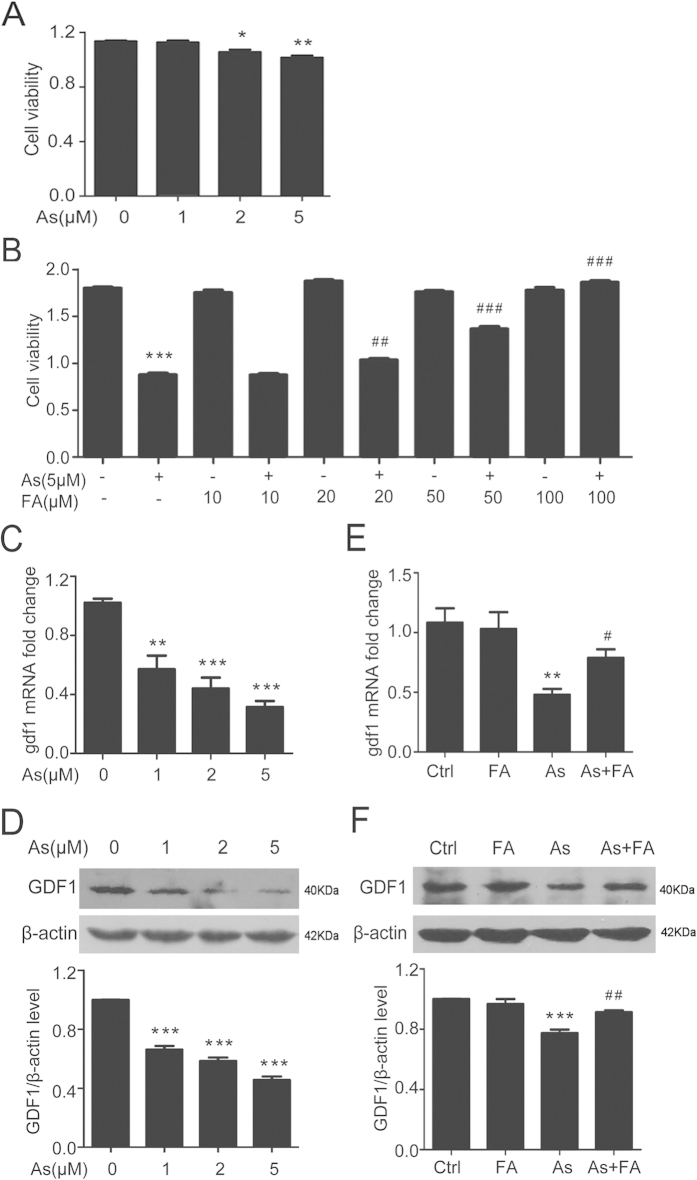
Folic acid up-regulates cell viability and the expression of GDF1 in arsenite-exposed HEK293ET cells. (**A**) Cell viability was assessed by CCK-8 proliferation assays in HEK293ET cells treated with different concentrations of arsenite. (**B**) Cell viability was assessed by CCK-8 proliferation assays in HEK293ET cells treated with arsenite, folic acid and their combination. (**C**) and (**D**) Expression of GDF1 was assessed by quantitative real-time PCR (C) and Western blot (D) in HEK293ET cells treated with different concentrations of arsenite. Quantification of GDF1 expression by densitometry. (**E**) and (**F**) Expression of GDF1 was assessed by quantitative real-time PCR (E) and Western blot (F) in HEK293ET cells treated with arsenite, folic acid and their combination. Quantification of GDF1 expression by densitometry. ^*^p < 0.05, ^**^p < 0.01, ^***^p < 0.001 vs control group, ^#^p < 0.05, ^##^p < 0.01 vs arsenic group. The data represent the mean±S.E. Ctrl, control; FA, folic acid; As, arsenite.

To further confirm the protective role of folic acid against arsenic toxicity, both cell viability and GDF1 expression were examined in HEK293ET cells. Cell viability decreased in response to arsenite at different concentrations (0, 1, 2, and 5 μM) in a dose-dependent manner. Incubation of cells with 2 or 5 μM arsenite significantly decreased cell viability compared with the control treatment (Fig. 5A). Conversely, folic acid treatment inhibited this decrease in a dose-dependent manner (Fig. 5B). When cells were co-treated with 5 μM arsenite and 20 μM folic acid, the cell viability was significantly increased compared with the group treated with arsenite alone. Furthermore, when cells were treated with 100 μM folic acid, the cell viability was almost completely restored (Fig. 5B). GDF1 mRNA and protein expression were reduced in a dose-dependent manner in arsenite-treated cells (Fig. 5C,D). However, folic acid supplementation markedly inhibited the decrease in GDF1 mRNA and protein levels (Fig. 5E,F).

### Folic acid reduces ROS levels and the expression of p66Shc in arsenite-exposed HEK293ET cells

**Figure 6:**
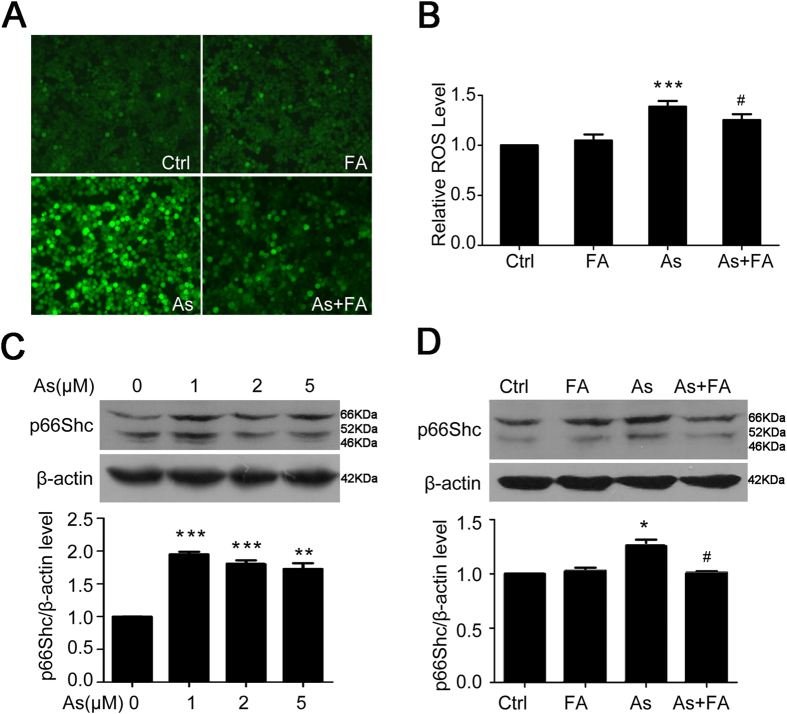
Folic acid reduces ROS levels and the expression of p66Shc in arsenite-exposed HEK293ET cells. (**A**) and (**B**) Levels of ROS were assessed by fluorescence microscope (**A**) and microplate reader (**B**) in HEK293ET cells treated with arsenite, folic acid and their combination. (**C**) Expression of p66Shc was assessed by Western blot in HEK293ET cells treated with different concentrations of arsenite. Quantification of p66Shc expression by densitometry. (**D**) Expression of p66Shc was assessed by Western blot in HEK293ET cells treated with arsenite, folic acid and their combination. Quantification of p66Shc expression by densitometry. ^*^p < 0.05, ^**^p < 0.01, ^***^p < 0.001 vs control group; ^#^p < 0.05 vs arsenic group. The data represent the mean ± S.E. Ctrl, control; FA, folic acid; As, arsenite.

Our previous research showed that p66Shc-linked redox imbalance mediated arsenite-induced embryonic retardation^13^,^14^. We investigated whether folic acid change the redox balance in arsenite-exposed HEK293ET cells. As shown in Fig. 6A,B, the level of subcellular ROS was significantly increased at 24 h after arsenite exposure compared to control treatment, whereas folic acid supplementation significantly reduced the production of ROS in arsenite-treated cells. As shown in Fig. 6C, p66Shc expression significantly increased in response to treatment with various doses of arsenite (0, 1, 2, and 5 μM). However, folic acid supplementation markedly inhibited the increase in p66Shc protein expression (Fig. 6D). These results showed that folic acid may play a protective role in arsenic-mediated cell toxicity by inhibiting the expression of p66Shc and subcellular oxidative stress.

### P66Shc knockdown decreases ROS levels and increases the expression of GDF1 in arsenite-exposed HEK293ET cells

**Figure 7:**
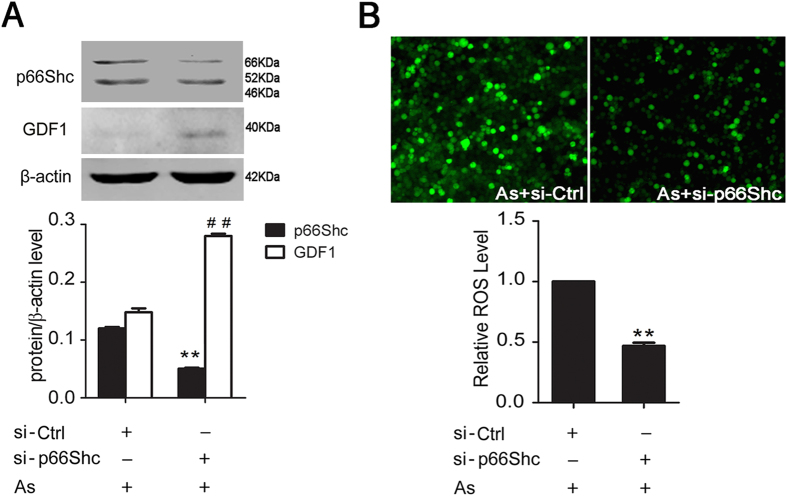
P66Shc knockdown decreases ROS levels and increases the expression of GDF1 in arsenite-exposed HEK293ET cells. (**A**) Expression of p66Shc and GDF1 in HEK293ET cells transfected with control or p66Shc siRNA in the presence of arsenite were assessed by Western blot. Quantification of p66Shc and GDF1 expression by densitometry. (**B**) Levels of ROS in HEK293ET cells transfected with control or p66Shc siRNA in the presence of arsenite were assessed by fluorescence microscope. ^**^p < 0.01, ^##^p < 0.01 vs control siRNA group. The data represent the mean ± S.E. As, arsenite; si-Ctrl, control siRNA; si-p66Shc, p66Shc siRNA.

We further assessed GDF1 expression and ROS levels in p66Shc knockdown cells in the presence of arsenite. HEK293ET cells were transfected with control or p66Shc-siRNA, incubated for 24 h, and then treated with arsenite for another 24 h. As shown in Fig. 7A, the knockdown efficiency was confirmed by Western blot. Knockdown of p66Shc attenuated the arsenite-induced down-regulation of GDF1 in HEK293ET cells. Correspondingly, the levels of subcellular ROS were significantly decreased in p66Shc knockdown cells compared with control cells (Fig. 7B). These results showed that knockdown of p66Shc inhibited the arsenite-induced down-regulation of GDF1 expression and the increase in the levels of ROS.

## Discussion

Epidemiological studies have confirmed that exposure to arsenic and its compounds can have adverse effects on human health by causing a series of cancers and cardiovascular and neurological diseases^27^. Recent studies reported that folic acid rescued lithium-, homocysteine- and Wnt3A-induced vertebrate cardiac anomalies^22^ and prevented complex cardiac defects after ethanol exposure during discrete cardiogenic events in zebrafish^28^. There is accumulating evidence that folic acid influences arsenic metabolism. FA treatment protected SWV/Fnn mouse embryo fibroblasts from sodium arsenite cytotoxicity^24^. The aim of the present study was to investigate the preventive role of folic acid against arsenic-induced embryo developmental toxicity. We observed that exposure to folic acid (50 μM) partly remitted the embryos’ low survival or obvious malformations caused by arsenic exposure during embryonic stages (48–96 hpf). In contrast, supplementation with higher concentrations of folic acid (100 μM) largely increased the survival rate and mitigated arsenic-caused changes in embryonic development, including delayed hatching, reduced growth and impairments in the neural and cardiac systems. We observed that the protective effect of folic acid decreased from 48 to 120 hpf. It may be associated with accumulation of arsenic in zebrafish^29^,^30^. Alternatively, folic acid may be incapable of recovering some signaling pathways altered by arsenic at early stage, which thereafter caused late developmental anomalies of zebrafish. These data enriched our knowledge of the influence of folic acid on arsenic-induced toxicity in embryonic development.

The GDF1 signalling pathway is required for left-right patterning in mice^31^. The phenotypes observed with the GDF1 loss-of-function mutation in mice are consistent with a model of disturbed left-right patterning with incomplete establishment of left-sided malformations seen in heterotaxy, including heart defects such as an abnormal atrium and ventricle^32^. Our previous study showed that the expression of Dvr1 was decreased in arsenite-treated embryos and that the over-expression of GDF1 rescued the cardiac and neural developmental defects caused by morpholino or arsenic in zebrafish embryos^26^. In this study, our data showed that folic acid rescued arsenic-induced embryonic developmental defects. Both real-time PCR and *in situ* hybridization showed that folic acid relieved the decrease in Dvr1 expression caused by arsenite treatment in zebrafish embryos. These data indicated that folic acid may antagonize arsenic-mediated developmental defects by up-regulating the expression of Dvr1/GDF1. Wnt/β-catenin signaling is implicated in cardiac left-right patterning and exhibits distinct and developmental stage-specific roles during cardiogenesis^33^. Linask *et al.* have showed that alcohol, lithium or homocysteine induction and folic acid protection of cardiac defects involved intimate control of Wnt/β-catenin signaling at a crucial time preceding, and during, early heart organogenesis^20^,^22^,^34^. This suggested that Dvr1/GDF1 may be a novel target of environmental effects on cardiogenesis and neurogenesis. It has been accepted that leftward fluid flow within a ciliated organ during development results in the release of left-right asymmetric cues into the left side of the body, which then initiates the Nodal cascade in the left lateral plate mesoderm to control the left-sided development^35^. Recent study showed that Wnt/β-catenin signaling regulates left-right asymmetric development of vertebrate embryos by regulating ciliated organ formation and function^36^. Peterson *et al.* reported that Dvr1 is responsible for enabling the transfer of a left-right signal from Kupffer’s vesicle (a ciliated organ in zebrafish) to lateral plate mesoderm^37^. Our previous study showed that Dvr1 and other signal molecules related to heart development were down-regulated in embryos treated with arsenite during gastrulation and overexpression of GDF1 partly rescued arsenic-mediated developmental defects^26^. Altered Wnt/β-catenin signaling has been reported after arsenic exposure^38^,^39^. It raises possibility that Wnt/β-catenin signaling may be also involved in arsenic induction and folic acid protection of developmental defects in zebrafish. The molecular network modulated by arsenic and folic acid needs to be further investigated.

Oxidative stress plays an important role in arsenic-induced toxicity. The toxic effects of arsenic are highly dependent on defence mechanisms in the body, i.e., the status and dietary intake of B-vitamins and antioxidants^40^. The direct antioxidant properties of folic acid have been observed *in vitro* and *in vivo*^23^,^41^. Our data showed that arsenite led to an increase in ROS in HEK293ET cells, while folic acid significantly decreased the level of ROS. Correspondingly, the expression of GDF1 was decreased in response to arsenite but increased upon co-treatment with arsenite and folic acid. These data implied that the expression of GDF1 was responsive to oxidative stress, which is consistent with the previous result that the expression of GDF1 was down-regulated by hydrogen peroxide^42^. P66Shc has been implicated as a major regulator of ROS production and intracellular oxidative stress responses. Our previous study showed that arsenite up-regulated the expression of p66Shc in preimplantation embryos, whereas the antioxidant NAC inhibited this effect^13^. In this study, we found that p66Shc knockdown inhibited the increase in subcellular ROS level and the down-regulation of GDF1 induced by arsenite. Folic acid inhibited the increase in p66Shc protein levels and the down-regulation of GDF1 expression in HEK293 cells caused by arsenite treatment. These findings indicated that folic acid enhanced the expression of GDF1 by decreasing p66Shc expression and subcellular ROS levels. SIRT1, a class III histone deacetylase, negatively regulates p66Shc expression through epigenetic chromatin modification^43^. Garcia *et al.* reported that deficiency in folate and other methyl donors increases birth defects and induces cardiomyopathy through altered methylation and acetylation of PGC-1α and decreased SIRT1 expression^44^. This implied that folic acid decreased p66Shc expression possibly by up-regulating SIRT1 expression. Both decreased p66Shc expression and antioxidant properties of folic acid contribute to the reduction of subcellular ROS levels. However, the mechanism by which oxidative-redox signals modulate the expression of GDF1 needs to be further investigated.

One identified important risk factor for arsenic-related health effects is biotransformation, which involves reduction reactions (bio-activation) and methylation (mainly detoxification)^45^. Arsenic is methylated to monomethylarsonic acid (MMA) and dimethylarsinic acid (DMA) via one-carbon metabolism, a biochemical pathway that is dependent on folic acid^46^. Growing experimental and population-based evidence indicates that folic acid supplementation enhances arsenic methylation, which may contribute to the protective effect of folic acid against arsenic-mediated embryo toxicity observed in this study^40^,^46^. The change in global DNA methylation is an important regulatory mechanism during early embryogenesis^47^. McGauqhey *et al.* reported that embryonic and terminal tissues have unique methylation signatures in CpG islands and repetitive sequences in zebrafish^48^. Arsenite could induce epigenetic alterations, specifically by altering patterns of DNA methylation^49^. Our previous study showed that arsenic-treated embryos displayed abnormal hypomethylation in the trunk and tail at early time points and global hypermethylation with morphogenetic abnormalities later on^25^. Folic acid is one of the most important dietary sources of methyl groups and plays a crucial role in the methylation of DNA and regulators of gene expression via S-adenosyl methionine (SAM)^50^. Linask *et al.* reported that cardiac defects rescued by folic acid most likely involve one-carbon metabolism and epigenetic mechanisms^34^. Lee *et al.* demonstrated that human and zebrafish utilize similar one-carbon pathways and abnormal folate metabolism results in cardiac defects such as linear heart tubes or incomplete cardiac looping in zebrafish^51^. Du *et al.* reported that Setdb2, a SET domain-containing protein possessing a potential histone H3K9 methyltransferase activity, controls convergence and extension movements during zebrafish gastrulation by transcriptional regulation of Dvr1^52^. This implied that the effects of folic acid on arsenic-induced developmental defects and Dvr1 expression may be partially explained by its influence on embryo epigenomics.

In conclusion, we found that folic acid supplementation rescued arsenite-induced developmental abnormalities by up-regulating the expression of Dvr1/GDF1. Arsenite induced p66Shc expression and subcellular ROS generation to down-regulate GDF1 expression, while folic acid antagonized these effects. The present study provides insight into the risk assessment and prevention of arsenic-mediated embryo toxicity. This work also provides insight into a novel mechanism of folic acid protection against arsenic-mediated embryo toxicity.

## Material and Methods

All methods were performed in accordance with the approved guidelines.

### Zebrafish maintenance and embryo preparation.

The wild-type AB strain and Tg (cmlc2:GFP) zebrafish (*Danio rerio*) were raised according to guidelines for zebrafish cultivation as described previously^25^. Group mating was routinely carried out during the first 30 min of the light period. Embryos were collected during the first hour of the light period of the 14:10 light:dark (L:D) cycle and incubated in egg water (distilled deionized water containing 60 μg/ml Instant Ocean^®^ salt) at 28.3 °C. All zebrafish studies were performed under protocols approved by the Institutional Animal Care and Use Committee of the National Research Institute for Family Planning at Beijing.

### Embryo treatment and morphological observations of development

Sodium arsenite and folic acid were purchased from Sigma-Aldrich, St. Louis, MO, USA. Both a 1 M stock solution of sodium arsenite and a 100 μM folic acid stock solution were prepared in egg water, and aliquots were dispensed into Eppendorf tubes for storage at −20 °C. Before each bioassay, stock solutions were warmed to room temperature (RT, 25 °C) and used to prepare the final test concentrations in egg water or high glucose Dulbecco’s modified Eagle’s medium (DMEM, HyClone).

According to the stages of embryonic development described previously^53^, normally developing embryos were selected under a stereomicroscope (Zeiss Lumar, V12) at the blastula stage (4 hours post-fertilization, hpf). To determine the effective folic acid concentration for the subsequent experiments, zebrafish embryos were assigned to experimental treatment groups at 4 hpf (n=50 individuals/group) and randomly subjected to graded concentrations (50 and 100 μM) of folic acid with or without 2 mM sodium arsenite to investigate the developmental toxicity until 120 hpf. One control group (egg water only) was designated for each exposure test. Each treatment was replicated three times on different days/spawns. To suppress pigment expression, 0.003% (w/v) 1-phenyl-2-thiourea (PTU, Sigma) was added to perform histochemical or immunochemical stain experiments; it was not necessary to add PTU when we observed the effects of chemical exposure on the embryonic development of zebrafish using a bright-field stereomicroscope^54^. The egg water was replaced daily with fresh folic acid solution with or without arsenite in each dish. The percentages of surviving, affected and hatched fish were calculated and plotted against hpf.

### Assessment of cardiovascular phenotype

The described embryos were removed from incubation, and the cardiovascular phenotype was observed with a stereomicroscope (Zeiss Lumar, V12) and a CCD camera (AxioCam MRc5) at 72 hpf. Abnormal phenotypes of heart looping were observed under the microscope using Tg (cmlc2:GFP) transgenic embryos for the ventricle and atrium at 48 hpf. Haemoglobin staining with o-dianisidine was performed as previously described^55^. Impaired circulation was defined by the presence of pooled erythrocytes and a low number of circulating erythrocytes. All phenotypes were judged objectively with comparison to the normal phenotype. The counts at each time point were completed separately by two independent observers and averaged.

### Whole-mount immunochemistry

Neural patterning throughout the embryo can be observed by immunostaining of acetylated α-tubulin (α-AT), which reacts generally with all axon tracts^56^, using the standard method as described previously. Briefly, embryos of 48 hpf were dechorionated, fixed with 4% PFA at RT for 1 h, washed in phosphate-buffered saline (PBS) with 0.1% Tween-20 (Amresco), and blocked with blocking buffer containing 10% normal goat serum (Zhongshan Biotech) and 2 mg/ml bovine serum albumin (BSA, Sigma) for 2 h at RT. The blocking buffer was then removed, and the embryos were incubated with primary antibody (α-AT antibody, Sigma) at 4 °C overnight. They were then washed and incubated with IgG-FITC (Zhongshan Biotech) rabbit anti-mouse secondary antibody for 2 h at RT. After rinsing the embryos with PBS, micrographs were taken using a fluorescence stereomicroscope (Zeiss Lumar, V12).

### Whole-Mount *In Situ* Hybridization (WISH)

The plasmid containing the second exon of Dvr1 was obtained as a kind gift from Dr. Lehmann (Department of Ophthalmology and Medical Genetics, University of Alberta, Canada). The Dvr1 RNA probe, which was generated from the Dvr1 plasmid after linearization, was synthesized and labelled with a digoxigenin UTP-NTP mix (Roche) by T7 RNA polymerase (Promega). The whole-mount *in situ* hybridization of embryos was performed as described previously^57^. Briefly, embryos used for *in situ* hybridization were anesthetized in tricaine and fixed with 4% PFA. The expression of Dvr1 in zebrafish embryos was detected using alkaline phosphatase-conjugated antibodies and visualized by 4-nitro blue tetrazolium (Promega) and 5-bromo-4-chloro-3-indolyl-phosphate (Promega) staining; then, embryos were mounted in glycerol and photographed with a dissecting microscope (Zeiss Lumar, V12).

### Cell culture and viability analysis

HEK293ET cells (human embryonic kidney 293 cells) were cultured in high glucose DMEM (HyClone) supplemented with 10% foetal bovine serum (FBS), 50 μg/ml penicillin and 50 μg/ml streptomycin at 37 °C in a humidified atmosphere of 5% CO_2_. The cells (5 × 10^4 ^cells per well) were seeded in 96-well plates; at 75% confluence, they were subcultured approximately every other day. To analyse the protective effects of folic acid on cytotoxicity induced by arsenite, HEK293ET cells were treated with graded concentrations (1, 2 and 5 μM) of sodium arsenite and graded concentrations (10, 20, 50 and 100 μM) of folic acid with or without 5 μM sodium arsenite. The viable cell mass was measured using a Cell Counting Kit-8 (CCK-8, Dojin Laboratories, Kumamoto) to count live cells by WST-8. After a 24 h treatment, HEK293ET cells were incubated for 1 h in 10 μM CCK-8 diluted in cell medium. Afterwards, the cell viability was quantified using a microplate reader (BioTek) at 450 nm after subtracting the appropriate blank values.

### Isolation of RNA and real-time quantitative RT-PCR analysis

Total RNA was extracted from zebrafish embryos at 6 hpf or from HEK293ET cells using TRIzol Reagent (Invitrogen) according to the manufacturer’s instructions. One microgram of total RNA was reverse transcribed to cDNA for PCR (TAKARA). DNAMAN software (version 7.0, LynnonBiosoft) was used to design specific forward and reverse primers. The primer pairs were designed to span introns to prevent amplification of any contaminating genomic DNA. The details of these fragments are shown in Table 1. Real-time PCR was performed on an ABI Prism 7500 sequence detection system (Applied Biosystems) with SYBR green fluorescent labels. Samples (20 μl final volume) contained the following: 1× SYBR green master mix (Applied Biosystems) and 5 pmol of each primer. Cycling parameters were as follows: an initial step of 3 min at 95 °C, followed by 40 cycles of 15 s at 95 °C and 1 min at 60 °C. Each sample in each group was measured in triplicate, and experiments were repeated at least three times. The quantification was normalized to the endogenous control, β-actin. All reactions were performed in triplicate. Relative expression was calculated using the Ct values provided by the manufacturer.

### Small interfering RNA (siRNA) transfection

P66Shc siRNA and non-silencing control siRNA were purchased from Invitrogen and transfected using Lipofectamine^TM^ 2000 (Invitrogen) according to the manufacturer’s instructions. The p66Shc siRNA sequences were sense 5′-AUGAGUCUCUGUCAUCGCUTT-3′ and antisense 5′-AGCGAUGACAGAGACUCAUTC-3′^58^. HEK293ET cells were seeded in 12-well plates and grown overnight to approximately 60% confluence. Cells were transfected with 50 pmol siRNA. At 24 h post-transfection, cells were exposed to arsenite for another 24 h, and proteins were extracted for Western blot analysis.

### Western blot analysis

Cells were washed with PBS and lysed with a cell lysis buffer containing 0.5% sodium deoxycholate, 0.1% SDS, 10 mM NaF, 0.2 mM Na_3_VO_4_, 1 mM PMSF and a protease inhibitor cocktail (Roche). The protein concentration was measured using a Bradford Protein Assay Kit (Bio-Rad). Equal amounts of protein were loaded into each lane for separation by 12% SDS-PAGE. The resolved proteins were transferred to nitrocellulose membranes (Millipore), which were incubated with β-actin (Sigma), GDF1 (Epitomics) and p66Shc (Santa Cruz) antibodies, subsequently incubated with secondary goat anti-rabbit or goat anti-mouse IgG antibodies conjugated to horseradish peroxidase (Zhongshan Biotech), and then detected by enhanced chemiluminescence (ECL, Millipore). All experiments were performed in triplicate.

### Measurement of ROS

To measure the quantity of ROS produced during treatment, the level of ROS in the HEK293ET cells was examined by DCFH-DA (Sigma) using a modified version of the described protocol^59^. After 24 h of arsenite exposure, HEK293ET cells were washed and incubated in serum-free medium containing 10 μM DCFH-DA. After a further 30 min of incubation at 37 °C, cells were washed with serum-free medium to remove residual dye and immediately imaged with an Inverted Fluorescence Microscope (Nikon TE-2000U) at 450–490 nm excitation and 515–565 nm emission. The ROS levels were also detected using a microplate reader at 488 nm excitation and 525 nm emission (BioTek). Four independent experiments were performed; the order in which the groups were processed was randomized each time.

### Statistical analysis

Statistical analysis was performed using SPSS software (version 18.0, SPSS Inc.) and Graph Pad Prism software (version 5, GraphPad software). All experiments were performed at least in triplicate, the measurements were recorded by observers who were blinded to the groups, and the results are expressed as the mean ± S.E. Differences between treatment groups were analysed by one-way analysis of variance (ANOVA) with post hoc analysis using Student’s t test. For all analyses, differences were considered statistically significant when P < 0.05.

## Additional Information

**How to cite this article**: Ma, Y. *et al.* Folic acid protects against arsenic-mediated embryo toxicity by up-regulating the expression of Dvr1. *Sci. Rep.* **5**, 16093; doi: 10.1038/srep16093 (2015).

## Figures and Tables

**Table 1:** Primers in real-time quantitative RT-PCR analysis.

Gene-primer ID	primer sequence
zebrafish-β-actin-F	5′-GCCTTCCTTCCTGGGTATGG-3′
zebrafish-β-actin-R	5′-CAGACGGAGTATTTACGCTCAG-3′
zebrafish-Dvr1-F	5′-TCAGCCCTGTTGCGTTCC-3′
zebrafish-Dvr1-R	5′-ACTCATCCACCACCATGTCTTC-3′
homo-β-actin-F	5′-GGCAGGATTGGATCATTG-3′
homo-β-actin-R	5′-CATTCAAACTCTCGCTCAG-3′
homo-GDF1-F	5′-AGATCAAGATCATTGCTCCT-3′
homo-GDF1-R	5′-CTCGTCATACTCCTGCTT-3′
